# Supplementary material for: Comparative Study of Biochar Modified with Different Functional Groups for Efficient Removal of Pb(II) and Ni(II)
Source: Int J Environ Res Public Health. 2022 Sep 6;19(18):11163. doi: 10.3390/ijerph191811163 (PMC9517685; doi:10.3390/ijerph191811163)
Supplement: Supplementary file 1 [file ijerph-19-11163-s001.zip › ijerph-1865042-supplementary.pdf]

Supporting Information

Comparative Study of Biochar Modified with Different Functional Groups for Efficient Removal of Pb(II) and Ni(II)

Chengcheng Liu [1,†], Jiaxin Lin [2,3,†], Haojia Chen [2,3,4], Wanjun Wang [2,3,*] and Yan Yang [1,2,3,4,*]

[1] School of Environmental and Safety Engineering, Changzhou University, Changzhou 213164, China
[2] School of Environmental Science and Engineering, Institute of Environmental Health and Pollution Control, Guangdong University of Technology, Guangzhou 510006, China
[3] Synergy Innovation Institute of GDUT, Shantou 515041, China
[4] Chemistry and Chemical Engineering Guangdong Laboratory, Shantou 515041, China
* Correspondence: wanjun@gdut.edu.cn (W.W.); yangyan1209@gdut.edu.cn (Y.Y.)
† These authors contributed equally to this work.

Figures

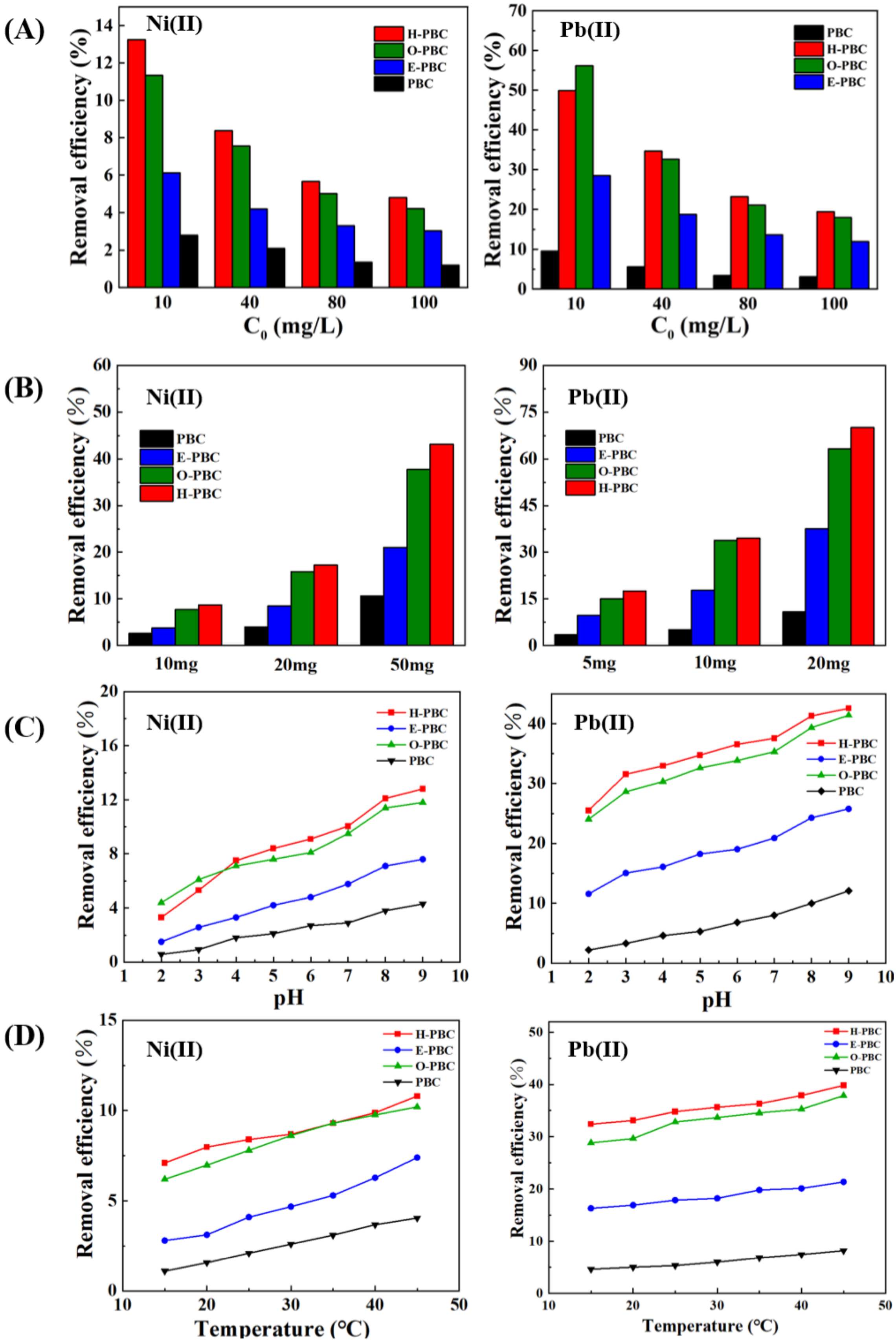
(A)
Ni(II)
H-PBC
O-PBC
E-PBC
PBC
Removal efficiency (%)
C0 (mg/L)
Pb(II)
(B)
10mg
20mg
50mg
5mg
(C)
pH
(D)
Temperature (°C)

Figure S1. (A) Effect of initial concentration on Ni(II) and Pb(II) removal efficiency. Experimental conditions: adsorbent dosage = 10 mg; pH = 5.0; temperature = 298.15 K; adsorption time = 9 h. **(B)** Effect of adsorbent dosage on Ni(II) and Pb(II) removal efficiency. Experimental conditions: [Ni(II)] and [Pb(II)] = 40 mg/L; pH = 5.0; temperature = 298.15 K; adsorption time = 9 h. **(C)** Effect of pH on Ni(II) and Pb(II) removal efficiency. Experimental conditions: adsorbent dosage = 10 mg; [Ni(II)] and [Pb(II)] = 40 mg/L; temperature = 298.15 K. **(D)** Effect of temperature on Ni(II) and Pb(II) removal. Experimental conditions: adsorbent dosage = 10 mg; [Ni(II)] and [Pb(II)] = 40 mg/L; pH = 5.0; adsorption time = 9 h.

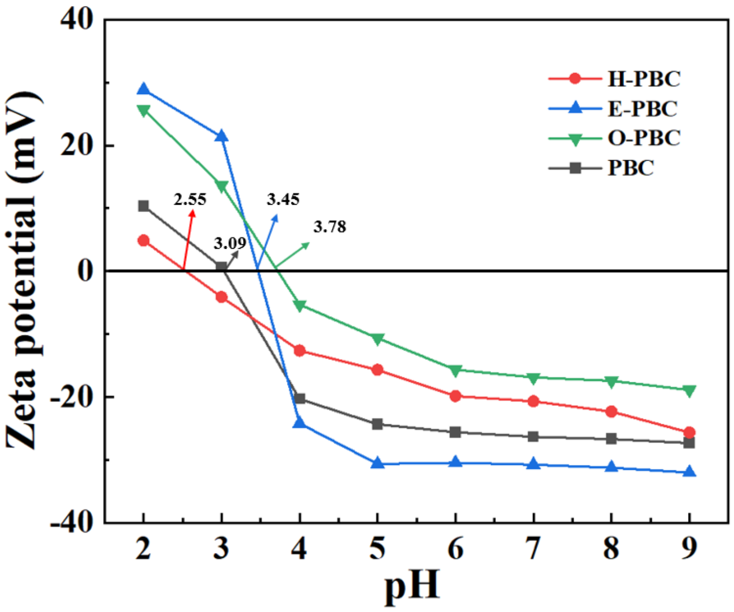

Figure S2. Zeta potential measurements

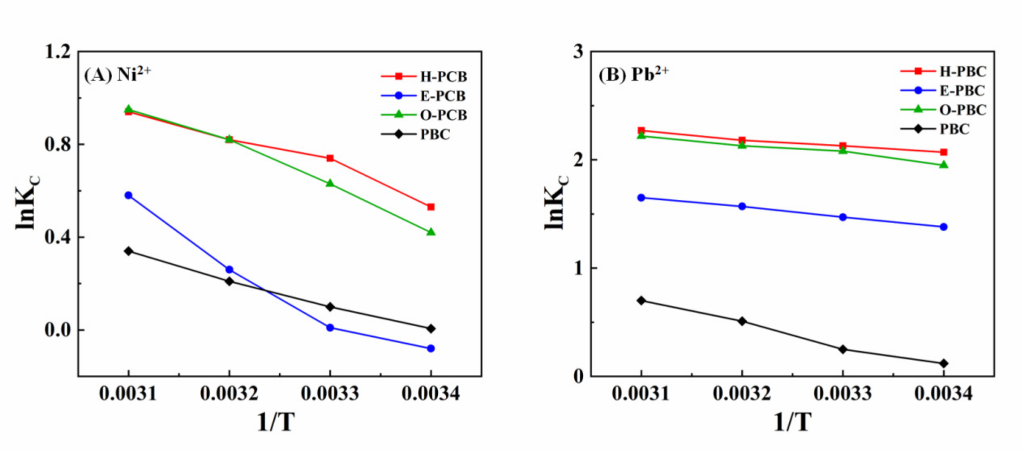

Figure S3. Plots of lnKc versus 1/T for the adsorption of (A) Ni(II) and (B) Pb(II) by PBC, H-PCB, E-PBC, and O-PBC.

Tables

Table S1. Main textural properties for PBC, H-PBC, O-PBC, and E-PBC.

Sample	BET surface area (m2/g)	Pore volume (cm^3/g)	Pore size (Å)
PBC	360.41	0.213	25.8
H-PBC	344.17	0.234	26.9
O-PBC	3.66	0.002	91.5
E-PBC	1.64	0.005	122.7

Table S2. Thermodynamic parameters for the adsorption of Ni^{2+} by PBC, H-PCB, E-PBC, and O-PBC at different temperatures.

Adsorbate	adsorbent	ΔH (kJ mol^{-1})	ΔS (J mol^{-1} K^{-1})	ΔG (kJ mol^{-1})			
				288.15 K	298.15K	308.15 K	318.1K
Ni (II)	PBC	6.79	23.6	−0.02	−0.25	−0.48	−0.72
	H-PBC	15.15	57.00	−1.27	−1.84	−2.41	−2.98
	E-PBC	15.98	56.00	−0.16	−0.72	−1.27	−1.83
	O-PBC	12.23	46.00	−1.02	−1.48	−1.94	−2.40

Table S3. Thermodynamic parameters for the adsorption of Pb^{2+} by PBC, H-PCB, E-PBC, and O-PBC at different temperatures.

Adsorbate	adsorbent	ΔH (kJ mol^{-1})	ΔS (J mol^{-1} K^{-1})	ΔG (kJ mol^{-1})			
				288.15 K	298.15K	308.15 K	318.1K
Pb (II)	PBC	8.62	31.00	−0.31	−0.62	−0.93	−1.24
	H-PCB	4.27	32.00	−4.95	−5.27	−5.59	−5.91
	E-PBC	5.85	34.00	−3.94	−4.28	−4.62	−4.96
	O-PBC	4.68	33.00	−4.83	−5.16	−5.49	−5.82

Table S4. Kinetic parameters for pseudo-first order and pseudo-second order models of Ni(II) adsorption by PBC, H-PBC, E-PBC, and O-PBC.

Samples	$Q_{e,exp}$ (mg/g)	Pseudo first-order			Pseudo second-order		
		q_{e1}(mg/g)	K_1(h^{-1})	R^2	q_{e2} (mg/g)	K_2(mg.$(gh)^{-1}$)	R^2
PBC	8.39	7.95	2.91	0.967	8.40	0.57	0.994
H-PBC	33.52	31.79	4.54	0.960	33.44	0.24	0.995
E-PBC	16.75	15.80	4.95	0.871	16.56	0.54	0.993
O-PBC	30.21	29.08	6.36	0.812	30.21	0.42	0.983

Table S5. Kinetic parameters for pseudo-first order and pseudo-second order models of Pb(II) adsorption by PBC, H-PBC, E-PBC, and O-PBC.

Samples	$Q_{e,exp}$ (mg/g)	Pseudo first-order			Pseudo second-order		
		q_{e1}(mg/g)	K_1(h^{-1})	R^2	q_{e2} (mg/g)	K_2(mg.(gh)$^{-1}$)	R^2
PBC	22.21	21.13	3.85	0.883	22.22	0.293	0.989
H-PBC	138.57	132.14	5.57	0.822	138.89	0.077	0.987
E-PBC	71.86	68.62	4.53	0.880	71.94	0.113	0.993
O-PBC	130.47	125.05	5.03	0.867	137.58	0.072	0.993

Table S6. Parameters fitted to Langmuir and Freundlich models of Ni(II) adsorption by PBC, H-PBC, E-PBC, and O-PBC.

	Langmuir			Freundlich		
	q_m (mg/g)	K_L(L.mg^{-1})	R^2	K_F	1/n	R^2
PBC	19.80	0.017	0.998	0.704	0.633	0.875
H-PBC	64.94	0.0295	0.996	4.225	0.548	0.885
E-PBC	47.17	0.0158	0.998	1.323	0.669	0.995
O-PBC	60.24	0.0262	0.999	3.493	0.563	0.978

Table S7. Parameters fitted to Langmuir and Freundlich models of Pb(II) adsorption by PBC, H-PBC, E-PBC, and O-PBC.

	**Langmuir**			**Freundlich**		
	**q_m (mg/g)**	**K_L(L.mg^{-1})**	**R^2**	**K_F**	**1/n**	**R^2**
PBC	38.31	0.037	0.999	3.368	0.491	0.981
H-PBC	243.90	0.0514	0.999	23.58	0.500	0.875
E-PBC	156.25	0.031	0.968	9.34	0.579	0.975
O-PBC	192.31	0.0935	0.997	31.907	0.403	0.939

Table S8. Comparison of various representative Ni^{2+} adsorbents.

**Years**	**Biomass feedstock**	**Finishing materials**	**Maximum adsorption capacity (mg.g^{-1})**	**Reference**
2017	Straw		25.06	[12]
	Rice husk		10.15	
2013	Figs	H_3PO_4	18.78	[7]
2017	Sludge	Mercaptan	52.40	[9]
2017	Sawdust	KOH	94.49	[11]
		$ZnCl_2$	19.36	
2020	Cactus	NaOH	44.35	[2]
2020	Rice bran	$Al(NO_3)_3$、$Mg(NO_3)_2$、$FeSO_4$	201.62	[6]
	Corn		19.80	This study
		H_3PO_4	64.94	
		EDTA	47.17	
		NaOH	60.24	

Table S9. Comparison of various representative Pb^{2+} adsorbents.

Years	Biomass feedstock	Finishing materials	Maximum adsorption capacity ($mg.g^{-1}$)	Reference
2014	Eggshell	ALG and PEI	344.8	[5]
2014	Bean shell		45.3	[3]
2014	Sawdust	H_3PO_4	80.65	[4]
2017	Banana peel	Ammonium sulfate persulfate	315.16	[14]
2018	Straw		134.68	[1]
2019	Shell	EDTA	129.31	[10]
2019	Peanut shells	H_3PO_4<br>PEI	353.4<br>214.0	[8]
2019	Lignin	PEI 和 CS_2	79.9	[13]
	Corn	 H_3PO_4<br>EDTA<br>NaOH	38.31<br>243.90<br>156.25<br>192.31	This study

References

1. Cao, Y., Xiao, W., Shen, G., Ji, G., Zhang, Y., Gao, C., and Han, L., Carbonization and ball milling on the enhancement of Pb(II) adsorption by wheat straw: competitive effects of ion exchange and precipitation, *Bioresour. Technol.*, 2019, vol. 273, pp. 70–76.
2. Choudhary, M., Kumar, R., and Neogi, S., Activated biochar derived from opuntia ficus-indica for the efficient adsorption of malachite green dye, $Cu^{(+2)}$ and $Ni^{(+2)}$ from water, *J. Hazard Mater.*, 2020, vol. 392, pp. 122441
3. Elaigwu, S.E., Rocher, V., Kyriakou, G., and Greenway, G.M., Removal of Pb^{2+} and Cd^{2+} from aqueous solution using chars from pyrolysis and microwave-assisted hydrothermal

carbonization of prosopis africana shell, *J. Ind. Eng. Chem.*, 2014, vol. 20, pp. 3467–3473.

4. Ghasemi, M., Naushad, M., Ghasemi, N., and Khosravi-fard, Y., A novel agricultural waste based adsorbent for the removal of Pb(II) from aqueous solution: kinetics, equilibrium and thermodynamic studies, *J. Ind. Eng. Chem.*, 2014, vol. 20, pp. 454–461.
5. Godiya, C.B., Liang, M., Sayed, S.M., Li, D., and Lu, X., Novel alginate/polyethyleneimine hydrogel adsorbent for cascaded removal and utilization of $Cu^{(2+)}$ and $Pb^{(2+)}$ ions, *J. Environ. Manage*, 2019, vol. 232, pp. 829–841.
6. Guo, Z., Chen, R., Yang, R., Yang, F., Chen, J., Li, Y., Zhou, R., and Xu, J. Synthesis of amino-functionalized biochar/spinel ferrite magnetic composites for low-cost and efficient elimination of Ni(II) from wastewater, *Sci. Total Environ.,* 2020, vol. 722, pp. 137822.
7. Gupta, V.K., Pathania, D., and Sharma, S., Adsorptive remediation of Cu(II) and Ni(II) by microwave assisted H_3PO_4 activated carbon, *Arab. J. Chem.*, 2017, vol. 10, pp. S2836–S2844.
8. Jiang, Q., Xie, W., Han, S., Wang, Y., and Zhang, Y., Enhanced adsorption of Pb(II) onto modified hydrochar by polyethyleneimine or H_3PO_4: an analysis of surface property and interface mechanism, *Colloid Surf. A: Physicochem. Eng. Asp.*, 2019, vol. 583, pp. 123962.
9. Li, J., Xing, X., Li, J., Shi, M., Lin, A., Xu, C., Zheng, J., and Li, R., Preparation of thiol-functionalized activated carbon from sewage sludge with coal blending for heavy metal removal from contaminated water, *Environ. Pollut.*, 2018, vol. 234, pp. 677–683.
10. Li, M., Wei, D., Liu, T., Liu, Y., Yan, L., Wei, Q., Du, B., Xu, W., EDTA functionalized magnetic biochar for Pb(II) removal: adsorption performance, mechanism and SVM model prediction, *Sep. Purif. Technol.*, 2019, vol. 227, pp. 10.
11. Nayak, A., Bhushan, B., Gupta, V., and Sharma, P., Chemically activated carbon from lignocellulosic wastes for heavy metal wastewater remediation: effect of activation conditions, *J. Colloid Interface Sci.*, 2017, vol. 493, pp. 228–240.
12. Shen, Z.T., Zhang, Y.H., McMillan, O., Jin, F., and Al-Tabbaa, A., Characteristics and mechanisms of nickel adsorption on biochars produced from wheat straw pellets and rice husk, *Environ. Sci. Pollut. Res.*, 2017, vol. 24, pp. 12809–12819.
13. Wang, Q., Zheng, C., Shen, Z., Lu, Q., He, C., Zhang, T.C., and Liu, J., Polyethyleneimine and carbon disulfide co-modified alkaline lignin for removal of Pb^{2+} ions from water,

Chem. Eng. J., 2019, vol. 359, pp. 265–274.

14. Zhou, N., Chen, H., Xi, J., Yao, D., Zhou, Z., Tian, Y., and Lu, X., Biochars with excellent Pb(II) adsorption property produced from fresh and dehydrated banana peels via hydrothermal carbonization, *Bioresour. Technol.*, 2017, vol. 232, pp. 204–210.
